# The Predicted Impact of Heart Disease Prevention and Treatment Initiatives on Mortality in Lithuania, a Middle-Income Country

**Published:** 2011-10-15

**Authors:** Thomas E. Kottke, Lina Jancaityte, Abdonas Tamosiunas, Vilius Grabauskas

**Affiliations:** HealthPartners, Inc; Kaunas University of Medicine, Kaunas, Lithuania; Kaunas University of Medicine, Kaunas, Lithuania; Kaunas University of Medicine, Kaunas, Lithuania

## Abstract

**Introduction:**

Disease-prevention programs compete with disease-treatment programs for scarce resources. This analysis predicts the impact of heart disease prevention and treatment initiatives for Lithuania, a middle-income Baltic country of 3.3 million people.

**Methods:**

To perform the analysis, we used data from clinical trials, the Lithuanian mortality registry, the Kaunas Monitoring of Trends and Determinants in Cardiovascular Disease (MONICA) register, Kaunas University Hospital and, when data from Lithuania were not available, the United States. We used the predicted reduction in all-cause mortality (as potentially postponable deaths) per 100,000 people aged 35 to 64 years as our outcome measure.

**Results:**

The number of potentially postponable deaths from risk factor prevention and management in the population without apparent heart disease is 556.3 (plausible range, 282.3-878.1). The number of potentially postponable deaths for people with stable heart disease is 280.4 (plausible range, 90.8-521.8), 7.0 with a public-access defibrillator program (plausible range, 3.8-8.9), and 119.0 for hospitalized patients (plausible range, 15.9-297.7).

**Conclusion:**

Although improving treatment of acute events will benefit individual patients, the potential impact on the larger population is modest. Only programs that prevent and manage risk factors can generate large declines in mortality. Significant reductions in both cardiac and noncardiac death magnify the impact of risk-factor prevention and management.

## Introduction

Lithuania, a country of 3.3 million in Northern Europe, lies on the eastern shore of the Baltic Sea, north of Poland, south of Latvia, and west of Belarus. It regained independence from the Soviet Union in 1990. Having a per capita gross national income (GNI) about one-quarter that of the United States, Lithuania is classified by the World Bank as an upper middle-income country (1). As with the United States, Canada, and the countries of Northern and Eastern Europe, coronary heart disease (CHD) is the leading cause of decreased life expectancy among middle-aged Lithuanians (2). Preventing chronic disease through lifestyle improvement is a priority in Lithuania, but privatization of health services, coupled with health care costs that are growing at a rate far greater than that of the GNI, could divert resources from disease prevention (3). Because prevention programs compete with treatment programs for scarce resources, policy makers need evidence that allocating resources to disease prevention programs will have the greatest effect on the population's burden of disease (personal communication between Lithuanian Minister of Health, Raimondas Šukys, and Vilius Grabauskas, November 9, 2010).

To document the potential impact of public health and clinical intervention strategies, we used a model that accounts for the entire population and is relevant to both public health and clinical interventions (4). With this model, we can evaluate existing or proposed interventions at any point along the heart disease continuum, from prevention of risk factors to treatment of advanced disease. In this article, we report the expected impact of interventions that are currently available or might be developed to prevent and treat heart disease for the Lithuanian population aged 35 to 64 years.

## Methods

We used the 2007 register of the Kaunas University Hospital Department of Cardiology for medical care data for hospitalized patients. Kaunas University Hospital, a facility with approximately 2,000 beds, is the teaching hospital for the Kaunas University of Medicine. Lacking Lithuanian data, we used data from the United States to estimate service-delivery rates to ambulatory patients (4). The MONICA research protocol was approved by the Kaunas Medical University institutional review board.

Our model divides the population into 3 prevalence pools: people with no apparent heart disease, people with symptomatic heart disease with a left ventricular ejection fraction (LVEF) greater than 35%, and people with symptomatic heart disease with an LVEF of 35% or less (4). This division takes into account the marked differences in mortality among the pools and acknowledges that different types of interventions are most efficacious in each of the 3 pools.

We categorized acute cardiac events as out-of-hospital cardiac arrest, acute or emergent events, and disease discovered in the ambulatory setting. We subdivided acute/emergent events into acute myocardial infarction with ST-segment elevation (STEMI) on electrocardiogram (ECG), acute heart failure with an LVEF of 35% or less, acute myocardial infarction without ST segment elevation (nSTEMI) on ECG, and unstable angina or other acute cardiac events. The model can account for any intervention that would be directed at anyone in the population who is at risk for heart disease, has stable chronic heart disease, or is experiencing an acute event, because each person must belong to 1 of the 3 pools, and all types of acute events are subsumed under the 3 broad categories of acute events.

We selected the number of potentially postponable deaths (PPD) as the outcome of interest for this analysis. A similar outcome has been used to estimate the source of the change in death rates from heart disease in the United States and several other countries (5-8). In this analysis, we calculated the number of deaths that can be prevented or postponed by improving risk factors or care as follows:

PPD = (expected mortality reduction when the intervention is implemented) x (mortality rate) x (1 – current implementation rate) x (number in population).

The analysis used the cumulative-relative-benefit approach of Mant and Hicks to calculate the joint effect of simultaneous interventions (9). This model has also been used to estimate the potential impact of improving care in the United States (4,10).

In our analysis, we used mortality from all causes, for several reasons. Most intervention trials report outcomes in terms of total mortality. Reducing the burden of heart disease risk reduces total mortality and deaths from other chronic diseases, and using total mortality as the endpoint eliminates the possibility that an intervention simply results in death from a different cause rather than reducing the probability of death.

### Prevalence and mortality data

We used the Kaunas Monitoring of Trends and Determinants in Cardiovascular Disease (MONICA) registry to estimate the prevalence of heart disease, and we used the Lithuanian death registry as the source of death rates for the subpopulation without heart disease (11). We did not have access to accurate all-cause mortality rates for the subpopulations with symptomatic heart disease with an LVEF greater than 35% and symptomatic heart disease with an LVEF of 35% or less. Therefore, we used the mortality rate ratios from Olmsted County, Minnesota (12,13). We estimated that the risk of death for people with heart disease and an LVEF greater than 35% is 2.84 times the risk of death for those without apparent heart disease, and the risk of death for people with heart disease and an LVEF of 35% or less is 11.02 times the risk of death for those without apparent heart disease.

We used published reports from clinical trials to estimate what the 1-year case-fatality rates for acute events would have been without the provision of modern treatments. On the basis of an epidemiologic observation (13), we estimated that the LVEF is 35% or less in half the cases of heart failure.

### Risk factor data

We used the 2001 Lithuanian MONICA registry risk factor data for the analysis (14). At least 200 men and 200 women had been screened in every 10-year age group (35-44 y, 45-54 y, and 55-64 y). The response rate for the survey was 62.4%. The register contains data from 625 men and 778 women. The survey included physical measurements (blood pressure, height, body weight, and hip and waist circumference), blood samples for serum cholesterol levels, and face-to-face interviews by the research staff for information on smoking.

**Smoking**

The MONICA smoking questionnaire included questions about smoking behavior (regular smoker, ex-smoker, never-smoker, occasional smoker), type of tobacco smoked (cigarettes, pipe, cigars), and number of cigarettes smoked per day. Participants who smoked at least 1 cigarette, cigar, or pipe per day were considered regular smokers.

**Blood pressure**

MONICA uses standard mercury sphygmomanometers for blood pressure measurement. Blood pressure was measured from the right arm of the subject after 5 minutes of rest in a sitting position. The fifth phase of Korotkoff sounds was recorded as diastolic BP. The mean of 2 readings was used. Arterial hypertension was defined as a systolic blood pressure level greater than 140 mm Hg, a diastolic blood pressure level greater than 90 mm Hg, or both. Participants who had taken antihypertensive drugs in the last 2 weeks were classified as hypertensive regardless of their blood pressure level.

**Sensitivity analysis**

We used 95% confidence intervals, when available, to define a plausible range for the estimates of mortality reduction attributable to an intervention. Otherwise, we used ±20% of the expected value as the plausible range. For the plausible range of the current level of implementation, we used ±20% of the observed value. For estimates of the number of deaths prevented or postponed, we defined the lower bounds of the plausible range by the following product: the lower bounds of the estimates for the population size, expected mortality rate without intervention, and expected effect of the intervention and the upper bound of the current rate of intervention. We defined the upper bounds of the plausible range of deaths prevented or postponed by the following product: the upper bounds of the estimates for the population size, expected mortality rate without intervention, and expected effect of the intervention and the lower bound of the current rate of intervention.

Because we provided the plausible range for each of the values used in the calculations, the reader can estimate the impact of the achievable level of implementation. For example, the PPD associated with adequate physical activity is calculated to be 303.6 (Table 1). If the reader were to believe that the prevalence of physically active individuals could be increased by only 20 percentage points rather than 81 percentage points, the new PPD would be 303.6 × 20/81, or 75.0. This PPD can be compared with the PPD for any other intervention. For example, the maximum plausible PPD associated with increasing the rate of primary angioplasty for all patients with a STEMI is 11.6  (Table 2).

## Results

### Prevalence pools

A Lithuanian population of 100,000 adults aged 35 to 64 years would comprise 92,842 people (95% CI, 91,410-94,274) with no apparent heart disease, 5,516 (95% CI, 4,413-6,619) with symptomatic heart disease with an LVEF greater than 35%, and 1,642 (95% CI, 1,314-1,970) with symptomatic heart disease with  an LVEF of 35% or less. We calculated that, during 1 year, 1,112 (95% CI, 876-1,355) people without apparent heart disease, 188 (95% CI, 120-271) with symptomatic heart disease and an LVEF greater than 35%, and 217 (95% CI, 139-312) with symptomatic heart disease with an LVEF of 35% or less would die.

### Acute events

We calculated that, in a given year, 168 people would have an out-of-hospital cardiac arrest, 152 would have a STEMI, 207 would be hospitalized for acute heart failure with an LVEF of 35% or less, and 233 would have an nSTEMI. Along with these events, 1,893 people would be hospitalized for unstable angina, and 253 would receive a new diagnosis of heart disease in the ambulatory setting (Table 3). The events associated with the greatest number of deaths during the ensuing year would be unstable angina followed by out-of-hospital cardiac arrest.

### Interventions in the prevalence pools

Among the 5 interventions associated with a lower risk of death or known to reduce death for people without apparent heart disease, the largest PPD is associated with population levels of adequate physical activity (Table 1). The analysis predicts that 556.3 deaths are potentially postponable if all 5 interventions were implemented simultaneously.

As with people without apparent heart disease, the largest PPD for patients with symptomatic heart disease and an LVEF greater than 35% is associated with physical activity. The composite PPD for this population pool is 114.8. The largest PPD for patients with symptomatic heart disease with an LVEF of 35% or less is also associated with physical activity. This PPD is followed by the PPD associated with device therapy, smoking rates, and use of spironolactone, a drug used to prevent sudden death in patients with cardiomyopathy. The composite potential PPD for this population pool is 165.6. The number of potentially postponable deaths for people with stable heart disease is 280.4 (plausible range, 90.8-521.8)

### Interventions at the time of acute events

On the basis of the assumption that community-wide placement of automated external defibrillators (AEDs) with bystander training does not exist, the PPD associated with bystander training and public access to AEDs is 7.0 (Table 2).

The largest PPD for patients who experience a STEMI is associated with the rate of primary angioplasty, followed by abstinence from tobacco. The composite PPD for STEMI is 8.6. Among the 7 interventions that have been shown to reduce mortality in patients hospitalized for heart failure with an LVEF 35% or less, the largest PPD is associated with cardiac rehabilitation followed by the use of statins. The composite PPD associated with this acute event is 16.5.

Among the 8 evidence-based interventions used to treat patients with an nSTEMI, the largest PPD is associated with immediate revascularization, followed by abstinence from tobacco. The composite PPD for nSTEMI is 11.2.

Among the 7 evidence-based treatments used to treat patients hospitalized for unstable angina and similar conditions, the largest PPD is associated with cardiac rehabilitation and smoking cessation. The composite PPD for unstable angina and similar conditions is 82.7. The combined PPD for all hospitalized patients is 119.0.

Among the 6 evidence-based interventions used to treat patients with heart disease discovered in the ambulatory setting, the largest PPDs are associated with prescription of beta blockers, followed by cardiac rehabilitation and smoking cessation. The composite PPD for heart disease discovered in the ambulatory setting is 3.5.

### Sensitivity analysis

The results of the calculations did not substantively change when we varied the size of the population pools, death rates, efficacy of intervention, and the current rates of intervention. The upper bound PPD for acute events (305.1) was less than the lower bound PPD for the prevalence pools (373.1). The upper bound of the PPD for immediate revascularization of all patients with a STEMI or an nSTEMI was 27.3. The lower bound of the PPD for dietary change (54.7) is twice this number, and the lower bound of the PPD for adequate physical activity is nearly 7 times this number. This means that, if dietary change were only as effective as the lower-bound estimate and only half of the population adopted an adequate diet, dietary change would still have the same population impact as immediate revascularization for all patients with STEMI or nSTEMI under the most optimistic assumptions about revascularization. By far the greatest opportunity to reduce mortality lies with improving risk profiles and care for people in the 3 prevalence pools (Figure).

**Figure.**
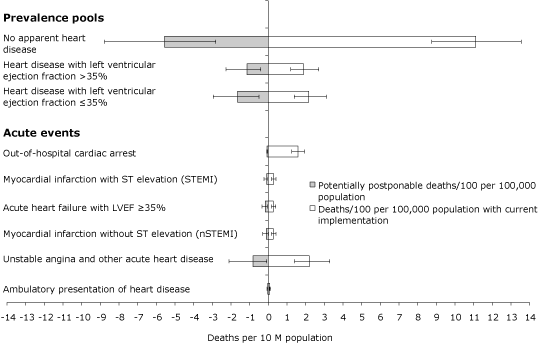
Open bars are deaths per 100 population with current level of implementation; shaded bars are potentially postponable deaths per 100 population. Error bars represent plausible range of estimate, defined as 1 standard deviation, if available; otherwise, 20% of the expected value. Abbreviation: LVEF, left ventricular ejection fraction.

**Table:** 

**Prevalence Pools**	**Potentially Postponable Deaths**	**Deaths at Current Levels of Treatment per 100,000 Population Aged 35 to 64 Years**
No apparent heart disease	556.3 (282.3 to 878.1)	1,112 (876 to 1,355)
Symptomatic heart disease with an LVEF >35%	114.8 (41.1 to 227.1)	188 (120 to 271)
Symptomatic heart disease complicated by an LVEF ≤35%	165.6 (49.7 to 294.7)	217 (139 to 312)
**Acute events**		
Out-of-hospital cardiac arrest	7.0 (3.8 to 8.9)	160 (126 to 193)
ST-segment elevation myocardial infarction	8.6 (2.3 to 23.2)	27 (18 to 41)
Acute heart failure due to LVEF ≤35%	16.5 (4.9 to 34.0)	27 (5 to 40)
Non ST-segment elevation myocardial infarction	11.2 (−0.4 to 30.3)	27 (17 to 41)
Unstable angina and other acute heart disease	82.7 (9.1 to 210.2)	220 (141 to 329)
Ambulatory presentation of heart disease	3.5 (1.2 to 7.4)	7 (5 to 11)

## Discussion

Our analysis indicates that interventions that would increase adoption of a low-risk lifestyle (not smoking, eating adequate fruits and vegetables, consuming foods high in omega-3 fatty acids, and obtaining adequate physical activity) and treatment of hypertension among people who are not known to have heart disease could potentially postpone more than one-third of all deaths in the Lithuanian population aged 35 to 64 years. Improving the delivery of care and improving lifestyles for ambulatory patients with heart disease could potentially postpone nearly 20% of all deaths. Optimizing care for people experiencing an acute event or with newly diagnosed heart disease could potentially postpone 8% of all deaths at most. Less favorable assumptions about prevalence, efficacy, mortality, and the ability to produce lifestyle changes do not substantively change the results. Risk-factor prevention and control and attention to improving ambulatory care for patients with heart disease is the strategy predicted to prevent or postpone the most deaths in the population; fewer than 10% of all deaths can be prevented or postponed by further improvements in care for patients hospitalized with heart disease.

This study has several limitations. Perhaps the most substantial is the lack of data specific to the Lithuanian population. Although the risk-factor data are highly reliable because they are based on carefully collected MONICA data, the only ambulatory care data available to us were from the United States. Clearly, Lithuanian data or data from a similar European state would have been more appropriate. Limiting the analysis to adults aged 35 to 64 when the highest mortality rates from heart disease occur in an older population segment is another limitation, but neither risk factor nor clinical care data exist for the older population. These shortcomings could all be corrected with more complete data from Lithuania; because the analysis is driven by an Excel spreadsheet, it can easily be updated with data from Lithuania or any other population. A more challenging shortcoming is the unknown extent to which newly developed interventions can lead to healthier lifestyles. Given the magnitude of the effect of lifestyle changes on death rates, interventions that would produce even modest movement toward healthier lifestyles would have a large effect on mortality.

Although the number of clinical trials to prevent and treat heart disease is very large, head-to-head comparisons of the expected effects of different interventions that address the burden of heart disease are nearly nonexistent. Using methods similar to those used in this analysis, Capewell et al calculated the expected impact of treating more people for coronary heart disease (10). However, their analysis addressed only medical and surgical interventions and limited the endpoint to death from heart disease rather than total mortality. An analysis that uses disease-specific death rates as the outcome variable underestimates the impact of risk-factor change. One of the authors of the current analysis (T.E.K.) published an analysis of the predicted effectiveness of various interventions if applied in the United States (4). As might be expected, the results were similar to those of the current analysis, because both reports used the same intervention data, and the major burden of disease in both countries is chronic disease.

The implications of this analysis extend beyond the borders of Lithuania. Cardiovascular disease — heart disease and stroke — is the leading cause of death in the world, and high-tech approaches to the problem are being aggressively marketed worldwide by technology and pharmaceutical companies. Particularly when specialist physicians are also advocating for large investments in high-tech solutions by pointing to improved outcomes in individual patients, policy makers may find it difficult to resist their arguments for large investments in medical technology and devices. We hope that access to analytic methods like the one used in this report can help make policy decisions more rational, evidence-based, and beneficial for population health.

## Figures and Tables

**Table 1:** Estimated Impact of Interventions Before or Between Acute Cardiac Events, Lithuania

Population Pool/Intervention	Proportion (95% CI)^a^		**PPD **per 100,000 Population (95% CI)

	**Expected Mortality Reduction in the Candidate Population**	**Current Level of Implementation^b^**	
**No Apparent Heart Disease**			
Improved diet	0.23^c^ (0.12-0.32)	0.40 (0.19-0.48)	153.5 (54.7-294.9)
Abstinence from tobacco	0.50^d^ (0.49-0.51)	0.81 (0.75-0.83)	105.7 (12.0-243.3)
Adequate physical activity	0.30^e^ (0.24-0.36)	0.09 (0.07-0.11)	303.6 (187.6-452.8)
Increase omega-3 fatty acid consumption	0.06^f^ (0.02-0.10)	0.41 (0.33-0.49)	42.0 (7.1-93.8)
Treat hypertension	0.25^g^ (0.20-0.30)	0.11 (0.29-0.44)	73.5 (44.6-111.2)
Composite potential			556.3 (282.3-878.1)
**Heart Disease With an LVEF >35%**			
Aspirin	0.20^h^ (0.16-0.24)	0.49 (0.39-0.59)	19.2 (7.9-39.5)
Beta blocker	0.23^i^ (0.15-0.31)	0.62 (0.50-0.74)	16.6 (4.6-42.3)
Statin	0.12^j^ (0.09-0.16)	0.41 (0.33-0.49)	13.3 (5.5-29.1)
Abstinence from tobacco	0.36^k^ (0.29-0.42)	0.71 (0.65-0.77)	19.8 (5.2-49.1)
ACE inhibitor	0.16^l^ (0.05-0.25)	0.66 (0.59-0.73)	10.2 (1.3-31.9)
Omega-3 fatty acids	0.20^m^ (0.06-0.33)	0.25 (0.20-0.30)	28.6 (5.1-71.4)
Adequate physical activity	0.42^n^ (0.25-0.71)	0.33 (0.26-0.40)	52.9 (18.2-141.4)
Composite potential			114.8 (41.1-227.1)
**Heart Disease With an LVEF ≤35%**			
Aspirin	0.20^h^(0.16-0.24)	0.55 (0.44-0.66)	19.5 (7.5-41.9)
Beta blocker	0.37^l^ (0.28-0.45)	0.85 (0.68-1.00)	12.1 (0.0-44.9)
ACE inhibitor	0.24^o^ (0.17-0.34)	0.85 (0.68-1.00)	7.8 (0.0-34.0)
Abstinence from tobacco	0.36^k^ (0.29-0.42)	0.71 (0.57-0.85)	22.6 (6.0-56.6)
Statin	0.12^j^ (0.09-0.16)	0.46 (0.28-0.55)	14.4 (5.6-31.6)
Spironolactone	0.30^p^ (0.18-0.40)	0.67 (0.54-0.80)	21.5 (4.9-57.9)
Adequate physical activity	0.63^q^ (0.16-0.83)	0.33 (0.26-0.40)	91.5 (13.4-190.7)
Device therapy with ICD plus biventricular pacemaker	0.43^r^ (0.20-0.60)	0.20 (0.16-0.24)	74.7 (21.1-157.3)
Composite potential			165.6 (49.7-294.7)

Abbreviations: CI, confidence interval; PPD, potentially postponable deaths; LVEF, left ventricular ejection fraction; ACE, angiotensin-converting enzyme; ICD, implantable cardioverter-defibrillator.

a The plausible range of the estimate is the 95% confidence interval if available and ±20% of the expected value when confidence intervals were not available.

b All current implementation estimates are from Kottke et al (4).

c Knoops et al (21).

d Doll and Peto (22).

e Andersen et al (23).

f Kottke et al (24).

g Antikainen et al (25).

h Anti-thrombotic Trialist Collaborative (26).

i Freemantle et al (27).

j Baigent et al (28).

k Critchley et al (29).

l Yusuf et al (30).

m GISSI investigators (31).

n Wannamethee et al (32).

o Flather et al (18).

p Pitt et al (33).

q Belardinelli et al (34).

r Lam et al (35).

**Table 2:** Estimated Impact of Interventions at the Time of an Acute Clinical Event, Lithuania

Clinical Event/Intervention	Proportion (95% CI) **a**		**PPD **per 100,000 Population (95% CI)

	**Expected Mortality Reduction in the Candidate Population**	**Current Level of Implementation^b^**	
**Out-of-Hospital Cardiac Arrest**			
Community-wide placement of automated external defibrillators with bystander training	0.05^c^ (0.04 to 0.06)	0.0	7.0 (3.8 to 8.9)
**ST-Segment Elevation Myocardial Infarction**			
Aspirin	0.20^d^ (0.16 to 0.24)	0.90 (0.72 to 1.00)	0.5 (0.0 to 2.8)
Beta blocker	0.04^e^ (−0.08 to 0.15)	0.94 (0.75 to 1.00)	0.1 (0.0 to 1.5)
ACE inhibitor	0.07^f^ (0.02 to 11)	0.94 (0.75 to 1.00)	0.1 (0.0 to 1.1)
Statins	0.12^g^ (0.09 to 0.16)	0.73 (0.58 to 0.88)	0.9 (0.2 to 2.7)
Primary angioplasty	0.50^h^ (0.40 to 0.60)	0.66 (0.53 to 0.79)	4.7 (1.5 to 11.6)
Abstinence from tobacco	0.36^i^ (0.29 to 0.42)	0.71 (0.57 to 0.85)	2.9 (0.8 to 7.4)
Cardiac rehabilitation	0.20^j^ (0.07 to 0.32)	0.90 (0.72 to 1.00)	0.5 (0.0 to 3.7)
Composite potential			8.6 (2.3 to 23.2)
**Acute Heart Failure Due to Left Ventricular Systolic Dysfunction**			
Aspirin	0.26^d^ (0.23 to 0.29)	0.53 (0.42 to 0.64)	3.3 (1.4 to 6.7)
Beta blockers	0.37^k^ (0.28 to 0.45)	0.84 (0.83 to 0.89)	1.6 (0.0 to 6.0)
Spironolactone	0.30^l^ (0.18 to 0.40)	0.69 (0.16 to 0.24)	2.5 (0.5 to 7.2)
ACE inhibitors	0.26^m^ (0.17 to 0.34)	0.86 (0.78 to 0.86)	1.0 (0.0 to 4.3)
Statins	0.20^n^ (0.16 to 0.24)	0.36 (0.29 to 0.43)	3.4 (1.6 to 6.9)
Abstinence from tobacco	0.36^i^ (0.29 to 0.42)	0.71 (0.65 to 0.77)	2.8 (0.7 to 7.3)
Cardiac rehabilitation	0.35^o^ (.08 to.54)	0.10 (.08 to.12)	8.5 (1.2 to 20.1)
Composite potential			16.5 (4.9 to 34.0)
**Non–ST-Segment Elevation Myocardial Infarction**			
Aspirin	0.20^d^ (0.16 to 0.24)	0.71 (0.85 to 0.96)	1.6 (0.4 to 4.2)
Beta blockers	0.04^e^ (−0.08 to 0.15)	0.87 (0.66 to 0.87)	0.1 (0.0 to 1.8)
Clopidogrel	0.07^p^ (−0.08 to 0.21)	0.33 (0.28 to 0.48)	1.3 (−0.8-6.3)
ACE inhibitors	0.07^f^ (0.02 to 0.11)	0.91(0.49 to 0.70)	0.2 (0.0 to 1.2)
IIb/IIIa inhibitors	−0.10^q^ (−0.29 to 0.14)	0.03 (0.17 to 0.49)	−2.6 (−4.8 to 5.5)
Immediate revascularization	0.37^r^ (0.23 to 0.48)	0.24 (0.44 to 0.46)	7.6 (2.8 to 15.7)
Statins	0.12^g^ (0.09 to 0.16)	0.62 (0.62 to 0.83)	1.2 (0.4 to 3.3)
Abstinence from tobacco	0. 36^i^ (0.29 to 0.42)	0.71(0.64 to 0.77)	2.8 (0.7 to 7.4)
Cardiac rehabilitation	0.20^j^ (0.07 to 0.32)	0.90 (0.24 to 0.36)	0.5 (0.0 to 3.6)
Composite potential			11.2 (−0.4 to 30.3)
**Unstable Angina and Heart Disease Other Than Myocardial Infarction and Heart Failure**			
Aspirin	0.20^d^ (0.16 to 0.24)	0.69 (0.55 to 0.83)	13.6 (3.9 to 35.4)
Beta blockers	0.04^e^ (−0.08 to 0.15)	0.78 (0.62 to 0.94)	1.9 (−0.7 to 18.6)
Clopidogrel	0.07^p^ (−0.08 to 0.21)	0.01 (0.01 to 0.01)	15.2 (−11.1 to 68.6)
ACE inhibitors	0.07^f^ (0.02 to 0.11)	0.85 (0.68 to 1.00)	2.3 (0.0 to 11.6)
Statins	0.12^g^ (0.09 to 0.16)	0.56 (0.45 to 0.67)	11.6 (4.1 to 29.1)
Abstinence from tobacco	0.36^i^ (0.29 to 0.42)	0.71 (0.57 to 0.85)	22.9 (6.0 to 59.8)
Cardiac rehabilitation	0.20^j^ (0.07 to 0.32)	0.30 (0.24 to 0.36)	30.7 (6.3 to 80.1)
Composite potential			82.7 (9.1 to 210.2)
**Ambulatory/Incidental Presentation^s^**			
Aspirin	0.25^d^ (0.23 to 0.27)	0.69 (0.63 to 0.75)	0.6 (0.2 to 1.3)
Beta blockers	0.23^e^ (0.15 to 0.31)	0.32 (0.26 to 0.38)	1.1 (0.4 to 2.5)
ACE inhibitors	0.13^t^ (0.06 to 0.19)	0.40 (0.32 to 0.48)	0.6 (0.1 to 1.4)
Statins	0.12^g^ (0.09 to 0.16)	0.65 (0.52 to 0.72)	0.3 (0.1 to 0.8)
Abstinence from tobacco	0.36^i^ (0.29 to 0.42)	0.71 (0.64 to 0.77)	0.8 (0.2 to 2.0)
Cardiac rehabilitation	0.20^j^ (0.07 to 0.32)	0.30 (0.24 to 0.36)	1.0 (0.2 to 2.7)
Composite potential			3.5 (1.2 to 7.4)

Abbreviations: CI, confidence interval; PPD, potentially postponable deaths; ACE, angiotensin-converting enzyme.

a The plausible range of the estimate is the 95% confidence interval if available and ±20% of the expected value when confidence intervals were not available.

b All implementation data are from the Kaunas University Hospital records.

c Hallstrom et al (36).

d Anti-thrombotic Trialist Collaborative (26).

e Freemantle et al (27).

f ACE Inhibitor Myocardial Infarction Collaborative Group (37).

g Baigent et al (28).

h Hartwell et al (38).

i Critchley et al (29).

j Taylor et al (39).

k Shibata et al (40).

l Pitt et al (33).

m Flather et al (18).

n Foody et al (41).

o Piepoli et al (42).

p Clopidigel in Unstable Angina (43).

q Ottervanger et al (44).

r Bavry et al (45).

s Kottke et al (4).

t Al-Mallah et al (46).

**Table 3:** Estimated Annual Number of Clinical Events in Population of 100,000 Adults Aged 35 to 64 Years, Lithuania

Clinical Event	**Number of Events**	**Case-Fatality Rate at Current Levels of Treatment**		**Deaths per Year at Current Levels of Treatment**

	n (Plausible Estimate Range)^a^	% (95% CI)		n (Plausible Estimate Range)^a^
Out-of-hospital cardiac arrest^b^	168^c^ (134-202)	0.95^d^ (0.94-0.96)	160 (126-193)	
STEMI	152 (122-182)	0.18^e^ (0.14-0.23)	27 (18-41)	
Acute heart failure due to LVEF ≤35%^f^	207 (166-248)	0.13^g^ (0.10-0.16)	27 (5-40)	
nSTEMI	233 (186-280)	0.12^g^ (0.09-0.15)	27 (17-41)	
Unstable angina/other heart disease^h^	1,893 (1,514-2,272)	0.12^i^ (0.09-0.15)	220 (141-329)	
Ambulatory/incidental presentation	253 (202-304)	0.03^j^ (0.02-0.04)	7 (5-11)	

Abbreviations: CI, confidence interval; STEMI, ST-segment elevation myocardial infarction; LVEF, left ventricular ejection fraction; nSTEMI, acute myocardial infarction without ST segment elevation.

a The plausible range of the estimate is the 95% CI, if available, and 20% of the expected value when confidence intervals were not available.

b Treatment of out-of-hospital cardiac arrest is defined as public access to automated external defibrillators with training.

c Centers for Disease Control and Prevention (15).

d Nichol et al (16).

e Baigent et al (17).

f On the basis of Olmsted County data (13), it is estimated that half of patients with heart failure have an LVEF ≤35%.

g Flather et al (18).

h Unstable angina/other heart disease is defined as one or more of ICD 9-CM codes 413, 414.1-414.9, 427.

i Peterson et al (19).

j American Heart Association (20).
